# Twelfth International Foamy Virus Conference—Meeting Report

**DOI:** 10.3390/v11020134

**Published:** 2019-02-01

**Authors:** Ottmar Herchenröder, Martin Löchelt, Florence Buseyne, Antoine Gessain, Marcelo A. Soares, Arifa S. Khan, Dirk Lindemann

**Affiliations:** 1 Institut für Experimentelle Gentherapie und Tumorforschung, Universitätsmedizin Rostock, 18057 Rostock, Germany; ottmar.herchenroeder@med.uni-rostock.de; 2 Department of Viral Transformation Mechanisms, Research Program Infection, Inflammation and Cancer, 69120 Heidelberg, Germany; m.loechelt@dkfz.de; 3 Unité d’épidémiologie et physiopathologie des virus oncogènes, Institut Pasteur, 75015 Paris, France; florence.buseyne@pasteur.fr (F.B.); antoine.gessain@pasteur.fr (A.G.); 4 CNRS UMR 3569, Institut Pasteur, 75015 Paris, France; 5 Departamento de Genética, Universidade Federal do Rio de Janeiro, Rio de Janeiro 21941-590, Brazil; masoares@inca.gov.br; 6 Laboratory of Retroviruses, Division of Viral Products, Center for Biologics Evaluation and Research, U.S. Food and Drug Administration, Silver Spring, MD 20993, USA; arifa.khan@fda.hhs.gov; 7 Institute of Virology, Medical Faculty “Carl Gustav Carus”, Technische Universität Dresden, 01307 Dresden, Germany; 8 Center for Regenerative Therapies Dresden, Technische Universität Dresden, 01307 Dresden, Germany

**Keywords:** foamy virus, spumaretrovirus, cross-species virus transmission, zoonosis, restriction factors, immune responses, FV vectors, virus replication, latent infection

## Abstract

The 12th International Foamy Virus Conference took place on 30–31 August 2018 at the Technische Universität Dresden, Dresden, Germany. The meeting included presentations on current research on non-human primate and non-primate foamy viruses (FVs; also called spumaretroviruses) as well as keynote talks on related research areas in retroviruses. The taxonomy of foamy viruses was updated earlier this year to create five new genera in the subfamily, *Spumaretrovirinae*, based on their animal hosts. Research on viruses from different genera was presented on topics of potential relevance to human health, such as natural infections and cross-species transmission, replication, and viral-host interactions in particular with the immune system, dual retrovirus infections, virus structure and biology, and viral vectors for gene therapy. This article provides an overview of the current state-of-the-field, summarizes the meeting highlights, and presents some important questions that need to be addressed in the future.

## 1. Introduction

The baroque city of Dresden, Germany and its modern biomedical research campus set the stage for the 12th International Foamy Virus Conference hosted and organized by Dirk Lindemann and his team. Based on a 24-year-old tradition since the first meeting, which was held in 1994 in London (Table 1), Dirk arranged an exciting scientific program on current aspects of foamy viruses (FVs), also known as spumaretroviruses.

FVs are the only type of viruses in the subfamily of *Spumaretrovirinae,* while all other retroviruses belong to the *Orthoretrovirinae*. A biannual meeting brings together most of the FV researchers from different international institutions to present their progress and discuss new developments, and also provides a platform for collaborations. The presentations at the Dresden meeting reflected the dynamics of the field: New and evolving topics, “old” questions being addressed with new technologies, and identification of priority topics that need attention in the coming years. The scientific and generational changes in the field were noted by first-time participating scientists in the meeting. Meanwhile, some topics addressed at past gatherings have now apparently been solved, for instance, the acceptance of the uniqueness of the FV replication strategy, which has led to the establishment of *Spumaretrovirinae* as a separate subfamily of retroviruses, followed by an updated taxonomy and nomenclature [1]. As another milestone, it had been the integrase protein of the prototype FV (PFV; designated as SFVpsc_huHSRV.13), which led to the first successful crystallization of a complete retroviral intasome complex and subsequent ultrastructural analyses of the active intermediates [2]. Thus, the FV integrase serves as the template structure for all retroviral/retroid element integrases. Due to their unique molecular biology, FVs may be considered relevant models to study yet unidentified principles of (retro-)virology. Ultimately, the taxonomic “upgrade” could draw other virologists’ attention to these viruses and eventually get more scientists involved in FV research.

In addition to presentations on many aspects of basic, applied, and translational biology of a constantly growing number of new and molecularly characterized FV isolates from diverse hosts, there were keynote presentations from related fields as well as opportunities that were provided for informal discussions and scientific exchange.

## 2. Summary of Scientific Sessions

The session chairs in consultation with the speakers developed the summaries below, which provide an overview of the current status and future directions for advancing the topic.

### 2.1. Epidemiology of Natural and Zoonotic Infections (Session Chair: Ottmar Herchenröder)

FVs are infectious agents that persistently infect primate, feline, bovine, and equine species as well as chiropterans (bats). Generally, FVs co-evolve with their host species. Having been an issue of debate in the early decades of FV research, we nowadays know that humans are not natural hosts of FVs. However, transmissions of simian FVs (SFV) to man are not uncommon in natural habitats shared by humans and non-human primates (NHPs), or in settings where the latter are held in captivity, such as in zoos or primate research centers. In contrast to the most prominent example of interspecies transmissions of retroviruses to man that initiated the worldwide AIDS epidemic caused by human immunodeficiency viruses (HIV), FVs are considered apathogenic in both their natural hosts and after interspecies transmissions.

In this session, researchers from the U.S., Brazil, and France gave further insights on the epidemiology of FVs and transmissions between species. Sue VandeWoude (Colorado State University, Fort Collins, CO, USA) reported on her group’s comparative epidemiologic surveys amongst mountain lions and domestic cats that share, in part, habitats in the Rocky Mountains foothills. Feline FVs (FFVs) are highly prevalent in these wild animals and, interestingly, interspecies transmissions from domestic cats to mountain lions were frequent as documented by sequence comparisons. Vice versa, FV from mountain lion (*Puma concolor*), was not seen in domestic cats, presumably, as the interaction between individuals of both species necessary for virus transmission may usually not allow the pet to walk away unscathed [3]. Marcelo Soares’ group (Universidade Federal do Rio de Janeiro, Rio de Janeiro, Brazil) had previously isolated and characterized FVs from a series of New World NHPs in Brazil. In Dresden, Marcelo’s co-worker, Cláudia Muniz, added new data on interspecies transmissions of SFVs from the “invading species”, Golden-headed lion tamarin (*Leontopithecus chrysomelas*), to the endangered local population of Golden lion tamarins *(Leontopithecus rosalia*) in the greater Rio de Janeiro area. This study also underlined the validity and robustness of oral swabs as a non-invasive method to achieve both species assignment and FV isolation and characterization [4]. Antoine Gessain (Institut Pasteur, Paris, France) presented a case-control study executed by French and Cameroonian scientists amongst hunters in Cameroon’s south-eastern rain forests that gave pause for thought whether simian FV-infections after interspecies transmissions always remain benign over the lifetime of the human recipient. The cases presented with deviations in several blood parameters in comparison to controls matching in residence, age, and social status [5]. Since clinical assessments did not differ between the cases and the controls, the data are reassuring for infected people. However, this study and most previous reports suffer from a strong bias as only apparently healthy individuals were studied. The audience’s attention was brought back to domestic animals by Magdalena Materniak-Kornas (National Veterinary Research Institute, Puławy, Poland), who analyzed co-infections with gammaherpesviruses and bovine FV (BFV) in cows suffering from post-partum metritis. By multiplex qPCR and Elisa techniques, the researchers found a good number of animals coinfected with both virus species [6]. Whether FVs have any influence on the clinical onset and burden of metritis in cattle remains speculative.

Altogether, some general beliefs hold true after this latest gathering of FV researchers. First, FV infections are common in numerous species, including livestock and many of those we humans conceive affection for, such as apes, monkeys, and of course cats. Second, FV infections remain in both the natural hosts or the foreign recipients after interspecies transmissions, apparently apathogenic as consented in the aftermath of the first FV conference (Table 1) [7]. More recent data, however, imply an association with hematologic abnormalities [5] and demand further research. Third, unlike many other retroviruses, FV distribution is a worldwide affair and those viruses appear phylogenetically more ancient than their pathogenic counterparts. As such, FVs may be considered as models to study in depth retrovirus biology and evolution.

The author of this section had a long time ago propagated FV isolates in Cf2Th, a cell line derived from canine thymus, which eventually resulted in the first complete sequence of a chimpanzee FV [8]. Cf2Th cells are very permissive for several FVs, although all canine species are considered free from non-endogenous retroviruses. With the surprises FV have entertained virologists over the last decades, he wonders, whether some day one will isolate a FV from man’s best companion, the dog. Similarly, it may be worth to look again whether any rodents harbor FVs.

### 2.2. Interactions of Foamy Viruses with the Immune System (Session Chairs: Antoine Gessain, Marcelo A. Soares)

Immunological aspects of infection by FV are still poorly characterized, both in naturally infected animals and in human infections after cross-species transmission. This scarcity of data includes both innate and acquired immunity, and several issues are still to be addressed in these areas.

What do we know about the immune responses directed against FVs? Several findings suggest strongly an efficient control of SFVs by the immune system in NHPs. These include the apparent lack of pathogenicity of SFVs in vivo, restriction of SFV RNA expression mainly to the oral cavity, and seemingly the gastrointestinal tract in some NHP species, as well as the very limited genetic variability in vivo. Furthermore, recent data indicate that FVs are susceptible to several restriction factors (see virus-host interactions Section 2.4), demonstrating the role of innate immunity in SFV infection, as well as, in some instances, in humans infected by SFVs of zoonotic origin [9,10]. In contrast, the control of SFV replication by innate immunity (antiviral molecules and cells), as well as adaptive immune responses (virus-specific antibodies and T lymphocytes), remain poorly characterized in NHP and even less in humans infected by an SFV.

The following questions remain poorly answered: (i) Can an immunodepression or a viral co-infection alter the pathogenicity of FVs in animal models and in natural infections? (ii) What are the molecular mechanisms by which FVs are efficiently sensed by innate immune cells? This will allow us to better understand how SFVs trigger an innate immune response, especially interferon (IFN) production, which has been demonstrated in blood plasmacytoid dendritic cells, a quite relevant model [11]. (iii) What is the respective part of each arm of the immune system in the modulation of FV infection and the apparent lack of pathogenicity of these viruses, especially in the context of zoonotic infection? (iv) What is the efficacy of antiviral activity of antibodies, especially the role and importance of neutralizing antibodies in vivo?

Answers to some of these questions were given at this meeting. Concerning immunosuppression and FV infection, a previous study has demonstrated that SIV coinfection in a macaque model led to expanded tissue tropism of SFV [12]. Conversely, SFV infection accelerated clinical progression, immunodeficiency, and viral load after SIV infection [13]. Thus, Cláudia Muniz and colleagues (Universidade Federal do Rio de Janeiro, Rio de Janeiro, Brazil) performed experiments in order to study the kinetics of SFV viral loads during immunosuppressive therapy, i.e., with tacrolimus, a drug commonly used to prevent graft rejection after transplantation. SFV DNA load was stable without major variation during the immunosuppression period in the two studied groups. The authors concluded that the immune mechanisms affected by tacrolimus, mostly T lymphocytes, were unable to modulate SFV replication at least during the chronic phase of the infection. In a South American NHP (*Brachyteles arachnoides*) with symptoms indicative of clinical immunodepression, likely due to infection by a simian type-D retrovirus (SRV), the same team demonstrated the presence of very high SFV DNA levels in the saliva and identified a new isolate, SFVbar [14]. Furthermore, transcriptome analysis from blood yielded SRV-related transcripts, but failed to identify SFVbar RNA, suggesting that SFVbar remained latent in PBMCs despite the immunocompromised status of the host.

Concerning antibodies, Florence Buseyne and colleagues (Institut Pasteur, Paris, France) demonstrated the presence of potent neutralizing antibodies in humans infected by zoonotic gorilla and chimpanzee FVs. Furthermore, Florence showed that the antibodies are directed against conserved epitopes located in the dimorphic domain of the surface envelope protein. The neutralization patterns provided evidence for persistent expression of viral proteins and a high prevalence of viral co-infection by two different viruses diverging in the *env* SU gene [15].

Mathilde Couteaudier and colleagues (Institut Pasteur, Paris, France) investigated whether plasma antibodies can inhibit SFV cell-to-cell transmission. Indeed, SFV transmission is considered highly cell-to-cell associated. Interestingly, she demonstrated that plasma samples from humans infected by zoonotic SFV, selected for their potent ability to neutralize cell-free virus, were unable to inhibit virus cell-to-cell spread.

In a second session devoted to the interaction between FV and the immune system, Florence Buseyne and colleagues (Institut Pasteur, Paris, France) characterized an immunodominant epitope located in the leader region of the SFV envelope protein (SFVpsc_huHSRV.13, aa 96–110) and recognized by the plasma antibodies of most SFV-infected hunters from Central Africa, similar to what has been recently described in FFV infections [16]. Whereas plasma from subjects infected with SFV derived from gorillas strongly recognized peptides corresponding to that envelope region of SFV from apes and other Old World primates, recognition was poor or absent to the respective peptides from SFV infecting more distant New World primates or from a specific African green monkey strain, suggesting evolutionary constraints in the adaptive immune responses observed. Whether the magnitude of such antibody responses can serve as proxies of overall adaptive immunity against SFV still remains to be determined.

By using FFV, Cláudia Muniz and coworkers (Universidade Federal do Rio de Janeiro, Rio de Janeiro, Brazil) had determined the effect of coinfections between FV and other retroviruses, in this case, the feline leukemia virus (FeLV) in domestic cats. Since FeLV causes immunodeficiency in their natural hosts, the feline model can also be used to understand the kinetics of FFV infection in the context of immunosuppresion. The investigators found that among FeLV/FFV-coinfected cats, those displaying an FeLV regressive infection (that tends to spontaneous clearance of the virus in the infected host), FFV replication and potential transmissibility is also reduced in the oral cavity, suggesting a synergistic interaction of both retroviruses as recently confirmed by Powers et al. [17]. On the other hand, FFV proviral loads in the peripheral blood did not differ between cats with or without clinical signs after FeLV infection, again highlighting a lack of association between FV replication and the general immunosuppressive status of the host, as pointed out in the studies conducted in NHP described above.

The last two presentations of the session addressed the role of innate immunity on FV infection in a very complementary manner. Jakob Weber and coworkers (Technische Universität Dresden, Dresden, Germany) showed the utility of the Phorbol 12-myristate 13-acetate (PMA)-differentiated monocytic cell line, THP-1, as a model system to study innate sensing in macrophages and monocytes, the likely first immune cell types to contact FV particles in infected tissues. The researchers found that incubation of these cells with PFV resulted in the induction of interferon-stimulated genes (ISGs). Furthermore, they demonstrated that PFV sensing is independent of Tas expression and integration of the proviral genome, but rather occurs at cell entry and requires access to the cytoplasm of the infected cell, with reverse-transcribed DNA being a likely recognized molecule. Maïwenn Bergez and colleagues (Paul-Ehrlich-Institut, Langen, Germany) showed that transduction of PFV vector particles into both THP-1 cells and primary human monocyte-derived dendritic cells induces the expression of the interferon regulatory factor 3 (IRF3)-dependent ISGs. Such induction was highly attenuated when RT-deficient viruses were used or azidothymidine was added during transduction, confirming that the newly reverse-transcribed PFV DNA acts as a pathogen-associated molecular pattern that stimulates the host cell innate immune system. The cytosolic sensors involved in the innate immune response to FV infection are yet to be determined.

### 2.3. Molecular and Cellular Biology of Foamy Viruses (Session Chair: Arifa S. Khan)

The development of advanced molecular tools and new generation biochemical reagents have enabled us to re-visit important questions regarding the biology and replication of FVs. Our current knowledge regarding the FV life cycle is mainly based on studies with SFVpsc_huHSRV.13, since this was initially believed to be a human FV (until it was sequenced and identified as a chimpanzee FV, which had infected a human by cross-species transmission). The information has been expanded based on FV isolates from other NHP species and extended by studies on FFVs and BFVs. The majority of the early work is based on laboratory virus strains. Although a few efforts are currently directed at studying the biology of natural FV isolates, most are from NHPs and only recently have also included New World monkeys. This research gap is further being addressed by studies investigating natural infections of FVs in bovine and feline species. Moreover, the availability of new and more sensitive assays that can investigate the different steps of FV replication, from infection/entry through integration/expression, resulting in release/exit, are important for understanding natural virus transmission and cross-species infections in humans by NHP FVs. To aid these goals, there is a need to develop reagents and functional assays for studying the molecular and cellular biology of naturally-occurring FV isolates. This session highlighted applications of new molecular tools and assays for investigating critical steps in spumaretrovirus replication.

Stefanie Richter from the Lindemann laboratory (Technische Universität Dresden, Dresden, Germany) reported on the adaptation of the fluorescent-based β-lactamase (BlaM) fusion assay [18] for FVs to obtain new insights about the kinetics and temperature requirements of FV Env-mediated fusion. The study used BlaM-containing SFVpsc_huHSRV.13 single-round vector particles habouring either SFVpsc_huHSRV.13 or SFVmcy_FV21 Env proteins and the Pac2 zebrafish embryonic cell line, which was previously thought to be resistant to FV Env-mediated entry [19]. Using Pac2 cells loaded with the fluorescent BlaM substrate CCF4-AM, the researchers demonstrated that this cell line was permissive for FV Env-mediated fusion. These results highlighted the potential of this approach for providing insight into the differences seen in the susceptibility of cells to FV infection.

Ivo Glück from the Lamb laboratory (Ludwig-Maximilians-Universität, München, Germany) presented on the application of the TrIC method [20] to track steps involved in the fusion process of single FV particles in live HeLa cells. Virus particles used in the study contained the viral envelope protein (Env) tagged with mCherry and the capsid protein (Gag) tagged with eGFP. By tracking the eGFP signal and locally cross-correlating the eGFP and mCherry intensities, the individual viral fusion events could be visualized as the capsid separated from the envelope. The analysis revealed a previously undetected intermediate step in the FV fusion process, in which the capsid and envelope signals are separated by approximately 300 nm, but remain tethered for an average of 7.1 min before full separation. An important next step is to identify the linking component that tethers the viral capsid to the envelope.

Martin Löchelt (German Cancer Research Center, Heidelberg, Germany) extended the previous identification of the chromatin binding site (CBS) in PFV Gag by functionally characterizing a highly conserved motif of the CBS of the Gag protein of FFV. It was demonstrated that the RYG residues in the QPQRYG motif are, in addition, essential during the early stages of infection after entry into the cytosol since mutagenesis of RYG abrogated the accumulation of Gag and proviral DNA in the nucleus and, subsequently, DNA integration into the host genome, similar to mutations within the chromatin binding. The results confirmed that chromatin binding by foamy virus Gag is a shared feature among FVs belonging to diverse genera and mediated by a conserved protein region located at the C-terminus of Gag, which is of prime importance for the provirus integration site. This motif likely influences the incoming virus capsid or its disassembly intermediates, but not newly synthesized Gag or its assembly products. The study, which was recently published [21] provides a strategy for comparing different FVs for mechanism and host cell conditions that can influence the efficiency of integration.

FV research using new assays and methodologies can be further enhanced and extended by the development of advanced technologies, such as next generation or high-throughput sequencing (NGS, HTS), which has emerged as a powerful tool that can help to investigate various aspects of virus biology and replication. In particular, transcriptomics and genomics studies using NGS could help determine the virus-host interactions that result in the seemingly general FV latency and limited expression seen in natural infections. The results from such studies may aid in predicting potential outcomes of FV infections in humans.

### 2.4. Mechanisms of Virus–Host Interactions: Cellular Antiviral Factors and Viral Antagonists (Session Chair: Florence Buseyne)

The interactions of FVs with their hosts were shaped by millions of years of coevolution [22]. FV have a ubiquitous tropism in vitro and numerous cross-species transmission events occurred in their evolutionary history. Thus, these viruses escape most constitutively expressed restriction factors. On the other hand, they are sensitive to the action of type I and type II IFN in vitro and are apparently apathogenic in vivo. Among the ISG products, TRIM5α, N-myc-interactor, IFP35, TRIM19, APOBEC, and tetherin were described to inhibit FV, while MxB and SAMHD1 had no action, and some discordant results are thus far unresolved. The currently open questions in the field, summarized after the former FV conference in 2016 [23], were about the role of restriction factors and IFN-induced genes in controlling FV gene expression in vivo in their natural hosts and in humans after cross-species transmission. Lack of knowledge on the induction and action of non-IFN antiviral actors (inflammasome, cytokines, and miRNA) were striking.

This conference provided new insights on four topics. Carsten Münk (Heinrich-Heine-Universität Düsseldorf, Düsseldorf, Germany) presented on APOBEC3s, which are potent inhibitors of FV. The viral Bet protein is the single antiviral factor antagonist described for FV. Its mechanism of action is unique: Bet prevents APOBEC3s incorporation into viral particles and thereby, provides protection of FVs, as well as lentiviruses, if Bet is expressed in trans, against APOBEC3s’ antiviral activity. Conversely, Carmen Ledesma-Feliciano reported in the FV vector session (see 2.6) that the feline immunodeficiency virus (FIV) Vif protein can replace the FFV Bet protein in vitro, but the chimeric virus was attenuated in vivo [24]. These in vivo attenuated phenotypes of Bet-deficient FV highlighted that additional Bet functions impacting FVs interaction with their hosts are waiting to be described.

Wenhu Cao from the Löchelt laboratory (German Cancer Research Center, Heidelberg, Germany) presented new results on bovine FV (BFV) miRNAs. BFV encodes miRNAs with a noncanonical biogenesis involving RNA polymerase III, generation of a single pri-miRNA, and its processing into three functional miRNAs. Cellular proteins were identified whose expression were suppressed by BFV miRNA and this was shown to result in repression of IFN-β and NF-κB pathways. Importantly, deletion of miRNA led to in vivo attenuation of BFV, confirming the major role of miRNAs in virus-host interactions.

Melissa Kane from the Bieniasz laboratory (The Rockefeller University, New York, NY, USA) presented new data based on results from the screening of nearly 500 human and macaque antiviral genes against 11 retroviruses, including the PFV [25]. The screen identified the ISG PHD finger protein 11 (PHF11) as a PFV-specific inhibitor. In vitro, PFV was unable to escape PHF11 restriction. PHF11 is involved in DNA damage signaling and DNA repair, and inhibits an early step in FV replication.

Arifa Khan (U.S. Food and Drug Administration, Silver Spring, MD, USA) presented the careful characterization of SFV-infected human A549 cell clones with various viral expression patterns, defined by viral DNA and RNA expression and production of infectious virions. Clones with fully latent and actively replicating SFV were analyzed for gene expression using next generation sequencing. Cells infected with different macaque SFV strains with widely different kinetics were included in the screen. Data overview showed diversity across strains in their ability to modulate IFN-signaling and other immune activation pathways.

Important questions and future directions were highlighted at the meeting. Data pointed to the uniqueness of FV (Bet, miRNA, and the PHF11) with elegant studies starting with molecular understanding of the activity of an FV component to in vivo assessment of its action. Such specificities open new molecular medicine applications, such as expression of heterologous regulatory RNA by BFV miRNA. The interest of high-throughput assays associated with well-validated in vitro models was evident and indeed, the PHF11 will not be identified by a hypothesis-driven strategy as early as with the comprehensive approach used [25]. In the future, new input is expected from modern biology approaches to identify cellular factors regulating viral replication and latency.

### 2.5. Structural Studies of Foamy Virus Proteins (Session Chair: Martin Löchelt)

Structural biology provides challenging insights into the molecular and atomic organization and underlying functions of proteins and molecular assemblies of high complexity. In addition, structural biology is a major tool in drug discovery and optimization and has enormous implications for translational research, medical care, and disease prevention. Under these perspectives, the current state-of-the-art of FV structural biology, focused in particular on integrase (IN) and the Gag structural proteins, was summarized in excellent reviews by Paul Lesbats (CNRS UMR5234, Bordeaux, France) and Jonathan Stoye (The Francis Crick Institute, London, United Kingdom).

FV Pol proteins share most of the conserved sequence motifs with the other retroviruses. However, their expression mode via a spliced transcript, resulting in the lack of a Gag-Pol fusion protein, most probably determines the uniqueness of FV particle assembly, genome incorporation, protein processing, capsid maturation, and reverse transcription. Another unique feature is the lack of processing of FV Pol precursor molecules to release the protease [3,22,26].

After a lot of unsuccessful attempts using other retroviruses, PFV IN was the first retroviral IN to be crystallized in its active form and as a high-molecular mass complex together with its target DNA [2]. Based on this structure and subsequent new data, highly divergent degrees of IN oligomerization in intasomes has been described for different retroviruses [27]. Here, PFV IN tetramers contrast other retroviral octo- and hexadecameric arrangements that make up the active intasomes. It is possible that the comparably low degree of PFV IN oligomerisation favored or even allowed its crystallization and thus, its structural analysis. Based on his experience in retroviral IN interactions with chromatin, Paul Lesbats described the interaction of the PFV chromatin-binding site with histones confirming and extending knowledge on C-terminal Gag residues involved in this process that contributes to FV integration site selection [28].

FV Gag and that of other retroviruses largely perform equivalent functions. However, several features, including the absence of genome-binding Cys-His motifs and a major homology region, the limited Gag processing at a very C-terminal site, and the essential requirement of N-terminal Gag and Env LP domains for particle release, are fundamentally different from that of other retroviruses [29]. The N-terminal and central domains of the unique FV Gag proteins are the focus of Jonathan Stoye’s past and current activities. Here, structural studies strongly target evolutionary issues related to retroviral capsid structures and their interaction with host-encoded restriction factors. Jonathan described structural analyses that identified two independent domains in a central part of FV Gag, which most probably derived from sequence duplications [30]. Surprisingly, these structures reveal a greater and unforeseen similarity to the Ty3 retrotransposon-derived mammalian protein ARC, than to conventional retroviral capsid proteins [31].

In summary, structural studies of FV Pol proteins have shown that they are highly related to those of other retroviruses and in the case of IN, even serve as model structures for the whole virus family. By contrast, although the amino-terminal and central domains of FV-Gag have been structurally characterized, the evolutionary origin and relationship with orthoretroviral Gag and exapted proteins found in mammalian genomes is the subject of current FV structural studies. Other FV proteins, most of them with unique features, have at large not been targets of high-resolution studies. Structural studies of FV Env are limited to a low-resolution (9 Å). Cryo-EM structure revealed the trimeric nature of the PFV Env gp80^SU^ spike [32] and the crystal structure an Env gp18^LP^ peptide bound to the N-terminal domain of PFV Gag [33]. Further high-resolution structural analyses of Env from two distinct serotypes of primate and feline FV SU could greatly foster the understanding of virus neutralization and the overlap with receptor binding, and may even support identification of the FV receptor(s). Finally, structural data on the non-canonical FV Tas transcriptional transactivator and the enigmatic Bet protein that counteracts APOBEC3-mediated restriction, but may also fulfil many additional functions, would fertilize further research on these essential and FV-specific proteins [34].

### 2.6. Development and Application of Foamy Virus Vector Systems (Session Chair: Dirk Lindemann)

Over the last 50 years human gene therapy has developed from fiction to become reality. After approval of the first gene therapy drugs several years ago, the last 24 months have seen a good number of new gene therapy drugs being approved and entering the market. The majority of them are based on viral gene ferries, which is also true for drugs currently being examined in gene therapy trials worldwide. This most likely reflects the fact that viruses may be considered as evolutionarily optimized nucleic acid delivery entities, although it took researchers decades to develop viral vector systems with optimized features of transduction efficiency, balanced expression control, and safety. Retroviral vectors are still the most frequent gene ferries used in gene therapy clinical trials and approved drugs. Gammaretroviral vectors based on murine leukemia virus were the first gene ferries being used in clinical trials. They were also employed in the SCID-X1 clinical trials around the turn of the century and were the first gene transfer tools with unequivocal therapeutic benefits for the patients. Lentiviral vectors based on HIV-1 were developed only later. Due to their ability to also efficiently transduce non-mitotic cells they have become more popular, which can be seen by the rising number of clinical trials using them as gene delivery tools.

The first vector systems based on FV were developed about 20 years ago [26,35]. FV have several natural features of a good candidate gene transfer vector, such as one of the largest retroviral genomes, an infectious DNA genome, a favorable integration site profile, and an extremely broad tropism. Most important is their apparent apathogenicity in both natural and zoonotic infections that renders them attractive for gene delivery. Furthermore, they have shown promising therapeutic results in preclinical studies using animal model systems [26,35]. However, so far, FV vectors or individual components of FV, such as the FV Env protein, have not been used in human gene therapy clinical trials.

Karol Budzik from the Russell laboratory (Mayo Clinic, Rochester, NY, USA) summarized his efforts to develop an FV platform for oncolytic virotherapy. SFVpve virus produced from a chimeric infectious molecular clone, which was derived from two chimpanzee FV strains (PAN1, PAN2), showed higher oncolytic activity in vitro than either parental strain or the PFV isolate in a variety of tumor cell lines. Furthermore, a U251 glioblastoma-derived reporter cell line was established and used to demonstrate in vivo replication of FV in a mouse model. This provides the basis for further examination of the tumoricidal potential of this new chimeric SFV strain or armed versions thereof in animal model systems.

Carmen Ledesma-Feliciano from the VandeWoude laboratory (Colorado State University, Fort Collins, CO, USA) presented a recently published study on the experimental infection in domestic cats with a replication-competent wild type and a novel FFV vaccine vector candidate expressing a truncated FFV-Bet/FIV-Vif fusion protein [24]. In vitro analysis in feline cells showed a requirement of FIV Vif expression without an FFV Bet N-terminal tag for efficient viral replication. In contrast, inoculation of immunocompetent domestic cats revealed a poor replication capacity of the vaccine vector in comparison to wild-type FFV. This suggests a yet uncharacterized role of FFV Bet for in vivo replication besides its anti-APOBEC activity.

Fabian Lindel from the Lindemann laboratory (Technische Universität Dresden, Dresden, Germany) reported on a novel PFV based vector system for largely transient expression of CRISPR/Cas9 genome editing tools in various target tissues. The system exploits the previously described natural feature of FV to encapsidate and efficiently transmit non-viral RNAs, which was exploited to express Cas9 fully transient in transduced cells. When combined with integration-deficient retroviral vectors harboring a U6 promoter-driven sgRNA, efficient gene inactivation was achieved in different target cell types. Additionally, the inclusion of a donor DNA template enabled efficient gene editing of reporter or cellular genes by homology directed repair mechanisms.

Finally, Jennifer Donau from the Valtink laboratory (Technische Universität Dresden, Dresden, Germany) summarized her work towards the establishment of a therapeutic proliferation stimulation strategy for primary human ocular tissues. She reported on the identification of viral and cellular proliferation promoting factors for immortalization of primary human corneal endothelial (CEC) or retinal pigment epithelial (RPE) cells upon stable transduction by lentiviral vectors pseudotyped by FV glycoproteins. Furthermore, she demonstrated that integration-deficient lentiviral vectors are not suited for transient growth stimulation in a therapeutic setting due to their residual non-viral mediated integration potential, which results in stable immortalization of primary tissues. At the end, she referred to her first attempts to achieve growth stimulation in these primary target tissues by transient expression of proliferation promoting factors using FV-mediated non-viral RNA gene transfer.

## 3. Keynote Lectures

The first keynote lecture was given by Welkin E. Johnson (Boston College, Chestnut Hill, MA, USA). Welkin presented an overview of a comparative study of ancient *env* genes of endogenous retroviral elements that provides insight into the co-evolution of retroviruses and their vertebrate hosts. He reviewed examples of potential cases of exaptations, whereby former endogenous retrovirus (ERV) *env* genes are preserved by purifying evolutionary selection and now provide cellular functions, for example, in the development (syncytins-mediated trophoblast fusion) or antiviral defense against extant viruses (ERV-mediated superinfection resistance). Furthermore, he summarized his and other people’s work on the origins and exaptive evolution of the ERV-Fc locus in mammals.

Frank Buchholz (Technische Universität Dresden, Dresden, Germany) delivered the second keynote lecture reviewing his and other researchers’ work on programmable nucleases and designer recombinases for genome surgery. He presented examples from his lab using CRISPR/Cas9 library screens to dissect driver mutations from passenger mutations in human cancer cell lines as an approach towards a personalized cancer therapy. Furthermore, he summarized the pioneering work of his and Joachim Hauber’s laboratory (Heinrich-Pette-Institut, Hamburg, Germany) on the broad-range anti-HIV-1 recombinase Brec1 and presented the latest results from experiments using humanized mouse models.

## 4. Conclusions

Two major goals were achieved by the 12th International Foamy Virus Meeting. First, bringing together most of the senior and junior scientists in the field with expertise in different disciplines for a scientific exchange and providing the opportunity for discussing ongoing, and initiating new collaborations. Second, attracting new people to the field, which was reflected by several first-time attendees, whose presentations strongly underlined the interdisciplinary character of the meeting and demonstrated the continued interest in this unique type of retrovirus. We enjoyed lively discussions after the individual presentations and in the breaks. We hope that the session summaries and highlights provided in this report illustrate that there is so much to be discovered and learned from FVs and will encourage interested researchers to join us at the 13th International Spumaretrovirus Conference, which is being planned for 2020 in Rio de Janeiro, Brazil. More information can be obtained by the conference host: Marcelo Soares masoares@biologia.ufrj.br.

## Figures and Tables

**Table 1:** Foamy virus conference history.

Year	Location	Key Findings Reported
1994	London, UK	Identification of “human foamy virus (HFV)” being the result of a zoonotic transmission of a chimpanzee FV Involvement of a defective HFV genome in viral latency Details of Tas-dependent HFV transcriptional control involving internal and LTR promoter
1997	Herzogenhorn, Germany	Full-length sequence of a feline FV isolate Reverse transcription during virus morphogenesis results in infectious FVs with a DNA genome First generation replication-deficient PFV vectors
1999	Gif-sur-Yvette, France	Characterization of an equine FV isolate FV glycoprotein-mediated subviral particle formation FV glycoprotein leader peptide-dependent viral particle release mechanism
2002	Atlanta, GA, USA	Application of 2nd generation replication-deficient PFV vector for hematopoietic stem cell gene transfer Viral genome-dependent FV Pol encapsidation mechanism
2004	Würzburg, Germany	Identification of FV Bet as inhibitor of the APOBEC restriction factors ESCRT-complex-dependent FV release First generation replication-deficient feline FV vector systems
2006	Seattle, WA, USA	Successful treatment of canine leukocyte adhesion deficiency by FV vector-mediated HSC gene transfer Centrosomal latency of incoming FV capsids in resting cells Identification of in vivo sites of FV replication in infected primates
2008	Heidelberg, Germany	CRM1-mediated nuclear export of unspliced FV RNAs PFV protease structure
2010	Argos, Greece	Host cells DDX6 protein involvement in PFV genome encapsidation Heparan sulfate as FV Env-dependent attachment factor
2012	Bethesda, MD, USA	Structure of the PFV Gag N-terminus in complex with FV Env LP peptides Innate sensing of FV by plasmacytoid dendritic cells Pseudotyping of FV vectors by heterologous viral glycoproteins
2014	Puławy, Poland	Discovery of BFV LTR encoded microRNAs Host cell Polo-like kinase interaction with PFV Gag involved in proviral integration Update of FV taxonomy and new nomenclature proposed
2016	Paris, France	Structure of the central domain of PFV Gag Genetic diversity of the *env* gene in zoonotic gorilla and chimpanzee FV strains
2018	Dresden, Germany	Identification of ISG PHF11 as cellular restriction factor of FVs Cross species dynamics of feline FV A FV vector system for transient expression of CRISPR/Cas genome engineering tools

## References

[1] Khan A.S., Bodem J., Buseyne F., Gessain A., Johnson W., Kuhn J.H., Kuzmak J., Lindemann D., Linial M.L., Lochelt M. (2018). Spumaretroviruses: Updated taxonomy and nomenclature. Virology.

[2] Hare S., Gupta S.S., Valkov E., Engelman A., Cherepanov P. (2010). Retroviral intasome assembly and inhibition of DNA strand transfer. Nature.

[3] Kehl T., Tan J., Materniak M. (2013). Non-simian foamy viruses: Molecular virology, tropism and prevalence and zoonotic/interspecies transmission. Viruses.

[4] Muniz C.P., Zheng H., Jia H., Cavalcante L.T.F., Augusto A.M., Fedullo L.P., Pissinatti A., Soares M.A., Switzer W.M., Santos A.F. (2017). A non-invasive specimen collection method and a novel simian foamy virus (SFV) DNA quantification assay in New World primates reveal aspects of tissue tropism and improved SFV detection. PLoS ONE.

[5] Buseyne F., Betsem E., Montange T., Njouom R., Bilounga Ndongo C., Hermine O., Gessain A. (2018). Clinical Signs and Blood Test Results Among Humans Infected With Zoonotic Simian Foamy Virus: A Case-Control Study. J. Infect. Dis..

[6] Materniak-Kornas M., Osinski Z., Rudzki M., Kuzmak J. (2017). Development of a Recombinant Protein-based ELISA for Detection of Antibodies Against Bovine Foamy Virus. J. Vet. Res..

[7] Weiss R.A. (1996). Reverse transcription. Foamy viruses bubble on [news]. Nature.

[8] Herchenröder O., Renne R., Loncar D., Cobb E.K., Murthy K.K., Schneider J., Mergia A., Luciw P.A. (1994). Isolation, cloning, and sequencing of simian foamy viruses from chimpanzees (SFVcpz): High homology to human foamy virus (HFV). Virology.

[9] Pinto-Santini D.M., Stenbak C.R., Linial M.L. (2017). Foamy virus zoonotic infections. Retrovirology.

[10] Rua R., Gessain A. (2015). Origin, evolution and innate immune control of simian foamy viruses in humans. Curr. Opin. Virol..

[11] Rua R., Lepelley A., Gessain A., Schwartz O. (2012). Innate sensing of foamy viruses by human hematopoietic cells. J. Virol..

[12] Murray S.M., Picker L.J., Axthelm M.K., Linial M.L. (2006). Expanded tissue targets for foamy virus replication with simian immunodeficiency virus-induced immunosuppression. J. Virol..

[13] Choudhary A., Galvin T.A., Williams D.K., Beren J., Bryant M.A., Khan A.S. (2013). Influence of naturally occurring simian foamy viruses (SFVs) on SIV disease progression in the rhesus macaque (Macaca mulatta) model. Viruses.

[14] Muniz C.P., Cavalcante L.T.F., Dudley D.M., Fedullo L.P., Pissinatti A., O’Connor D.H., Santos A.F., Soares M.A. (2018). First Complete Genome Sequence of a Simian Foamy Virus Infecting the Neotropical Primate Brachyteles arachnoides. Microbiol. Resour. Announc..

[15] Lambert C., Couteaudier M., Gouzil J., Richard L., Montange T., Betsem E., Rua R., Tobaly-Tapiero J., Lindemann D., Njouom R. (2018). Potent neutralizing antibodies in humans infected with zoonotic simian foamy viruses target conserved epitopes located in the dimorphic domain of the surface envelope protein. PLoS Pathog..

[16] Mühle M., Bleiholder A., Löchelt M., Denner J. (2017). Epitope Mapping of the Antibody Response Against the Envelope Proteins of the Feline Foamy Virus. Viral. Immunol..

[17] Powers J.A., Chiu E.S., Kraberger S.J., Roelke-Parker M., Lowery I., Erbeck K., Troyer R., Carver S., VandeWoude S. (2018). Feline Leukemia Virus (FeLV) Disease Outcomes in a Domestic Cat Breeding Colony: Relationship to Endogenous FeLV and Other Chronic Viral Infections. J. Virol..

[18] Jones D.M., Padilla-Parra S. (2016). The beta-Lactamase Assay: Harnessing a FRET Biosensor to Analyse Viral Fusion Mechanisms. Sensors.

[19] Stirnnagel K., Lüftenegger D., Stange A., Swiersy A., Müllers E., Reh J., Stanke N., Grosse A., Chiantia S., Keller H. (2010). Analysis of prototype foamy virus particle-host cell interaction with autofluorescent retroviral particles. Retrovirology.

[20] Dupont A., Stirnnagel K., Lindemann D., Lamb D.C. (2013). Tracking image correlation: Combining single-particle tracking and image correlation. Biophys. J..

[21] Wei G., Kehl T., Bao Q., Benner A., Lei J., Lochelt M. (2018). The chromatin binding domain, including the QPQRYG motif, of feline foamy virus Gag is required for viral DNA integration and nuclear accumulation of Gag and the viral genome. Virology.

[22] Rethwilm A., Bodem J. (2013). Evolution of foamy viruses: The most ancient of all retroviruses. Viruses.

[23] Buseyne F., Gessain A., Soares M.A., Santos A.F., Materniak-Kornas M., Lesage P., Zamborlini A., Löchelt M., Qiao W., Lindemann D. (2016). Eleventh International Foamy Virus Conference-Meeting Report. Viruses.

[24] Ledesma-Feliciano C., Hagen S., Troyer R., Zheng X., Musselman E., Slavkovic Lukic D., Franke A.M., Maeda D., Zielonka J., Munk C. (2018). Replacement of feline foamy virus bet by feline immunodeficiency virus vif yields replicative virus with novel vaccine candidate potential. Retrovirology.

[25] Kane M., Zang T.M., Rihn S.J., Zhang F., Kueck T., Alim M., Schoggins J., Rice C.M., Wilson S.J., Bieniasz P.D. (2016). Identification of Interferon-Stimulated Genes with Antiretroviral Activity. Cell Host Microbe..

[26] Lindemann D., Rethwilm A. (2011). Foamy virus biology and its application for vector development. Viruses.

[27] Engelman A.N., Cherepanov P. (2017). Retroviral intasomes arising. Curr. Opin. Struct. Biol..

[28] Lesbats P., Serrao E., Maskell D.P., Pye V.E., O’Reilly N., Lindemann D., Engelman A.N., Cherepanov P. (2017). Structural basis for spumavirus GAG tethering to chromatin. Proc. Natl. Acad. Sci. USA.

[29] Müllers E. (2013). The foamy virus Gag proteins: What makes them different?. Viruses.

[30] Ball N.J., Nicastro G., Dutta M., Pollard D.J., Goldstone D.C., Sanz-Ramos M., Ramos A., Müllers E., Stirnnagel K., Stanke N. (2016). Structure of a Spumaretrovirus Gag Central Domain Reveals an Ancient Retroviral Capsid. PLoS Pathog..

[31] Taylor W.R., Stoye J.P., Taylor I.A. (2017). A comparative analysis of the foamy and ortho virus capsid structures reveals an ancient domain duplication. BMC Struct. Biol..

[32] Effantin G., Estrozi L.F., Aschman N., Renesto P., Stanke N., Lindemann D., Schoehn G., Weissenhorn W. (2016). Cryo-electron Microscopy Structure of the Native Prototype Foamy Virus Glycoprotein and Virus Architecture. PLoS Pathog..

[33] Goldstone D.C., Flower T.G., Ball N.J., Sanz-Ramos M., Yap M.W., Ogrodowicz R.W., Stanke N., Reh J., Lindemann D., Stoye J.P. (2013). A Unique Spumavirus Gag N-terminal Domain with Functional Properties of Orthoretroviral Matrix and Capsid. PLoS Pathog..

[34] Lukic D.S., Hotz-Wagenblatt A., Lei J., Rathe A.M., Mühle M., Denner J., Münk C., Löchelt M. (2013). Identification of the feline foamy virus Bet domain essential for APOBEC3 counteraction. Retrovirology.

[35] Trobridge G.D., Horn P.A., Beard B.C., Kiem H.P. (2012). Large animal models for foamy virus vector gene therapy. Viruses.

